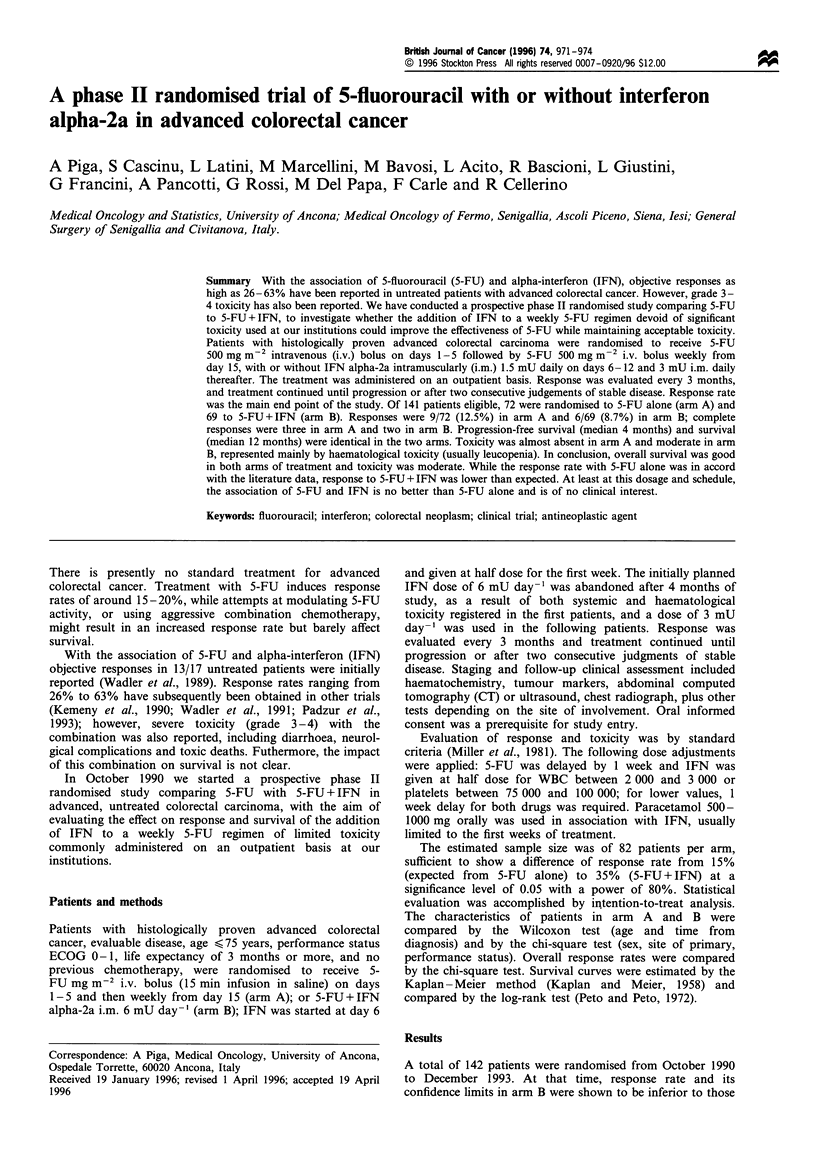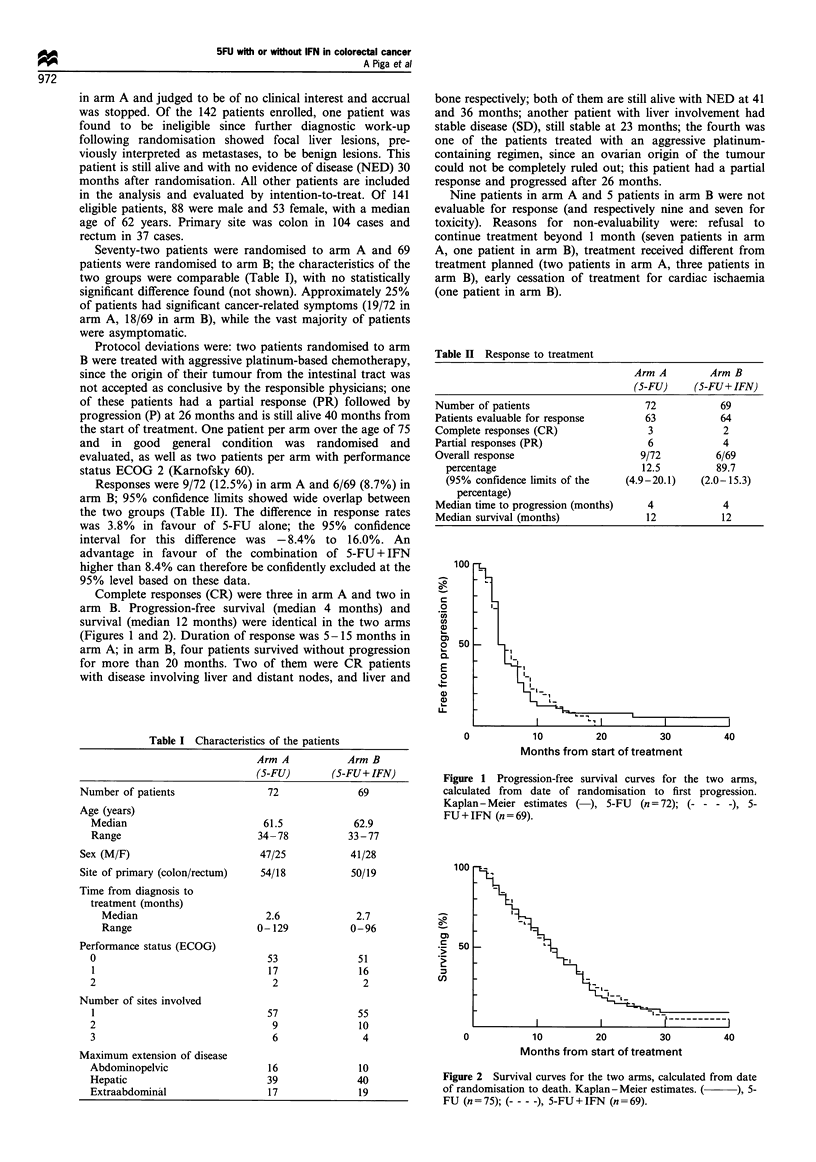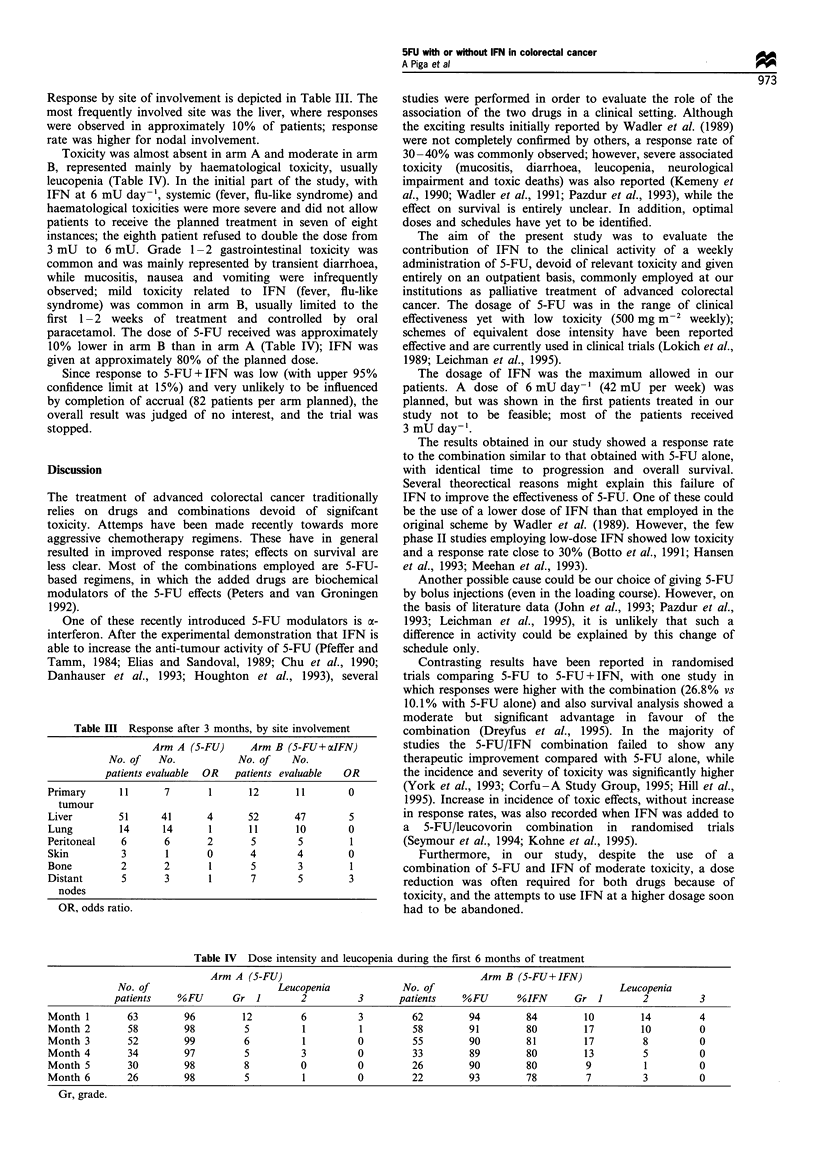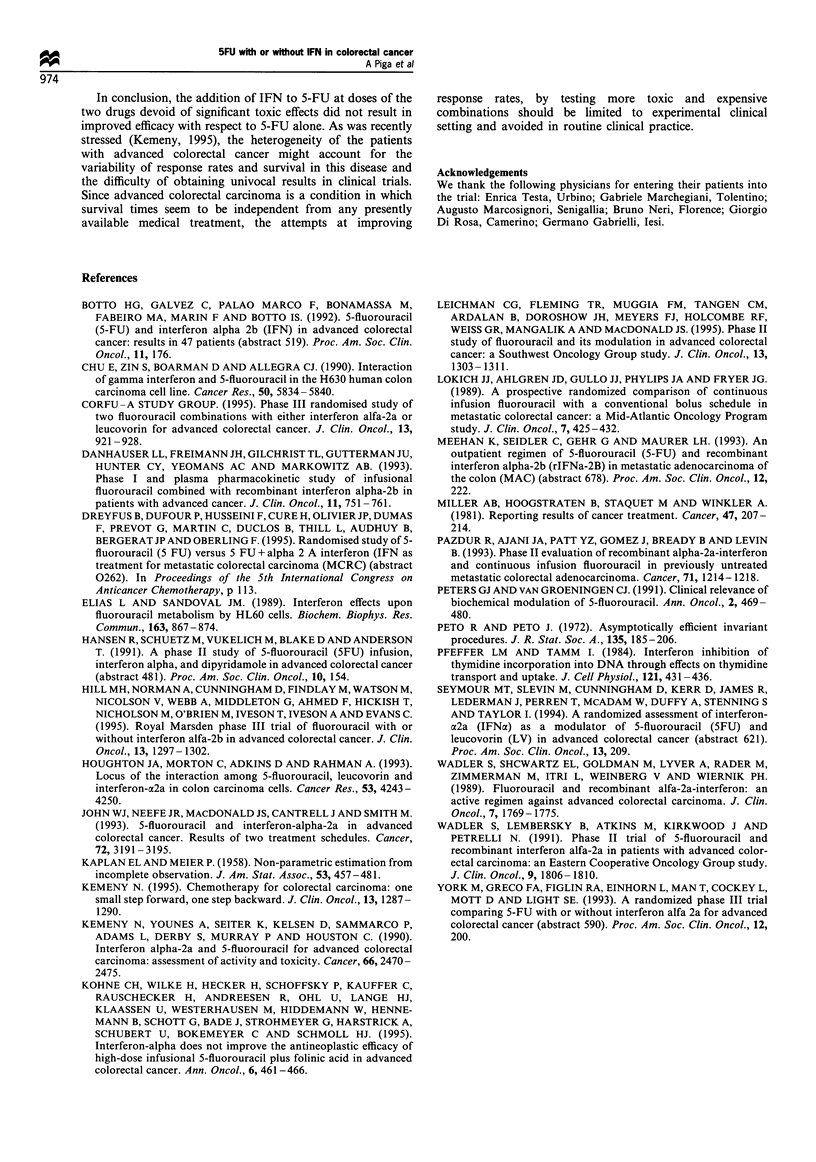# A phase II randomised trial of 5-fluorouracil with or without interferon alpha-2a in advanced colorectal cancer.

**DOI:** 10.1038/bjc.1996.467

**Published:** 1996-09

**Authors:** A. Piga, S. Cascinu, L. Latini, M. Marcellini, M. Bavosi, L. Acito, R. Bascioni, L. Giustini, G. Francini, A. Pancotti, G. Rossi, M. Del Papa, F. Carle, R. Cellerino

**Affiliations:** Medical Oncology and Statistics, University of Ancona, Italy.

## Abstract

With the association of 5-fluorouracil (5-FU) and alpha-interferon (IFN), objective responses as high as 26 63% have been reported in untreated patients with advanced colorectal cancer. However, grade 3-4 toxicity has also been reported. We have conducted a prospective phase II randomised study comparing 5-FU to 5-FU + IFN, to investigate whether the addition of IFN to a weekly 5-FU regimen devoid of significant toxicity used at our institutions could improve the effectiveness of 5-FU while maintaining acceptable toxicity. Patients with histologically proven advanced colorectal carcinoma were randomised to receive 5-FU 500 mg m-2 intravenous (i.v.) bolus on days 1-5 followed by 5-FU 500 mg m-2 i.v. bolus weekly from day 15, with or without IFN alpha-2a intramuscularly (i.m.) 1.5 mU daily on days 6-12 and 3 mU i.m. daily thereafter. The treatment was administered on an outpatient basis. Response was evaluated every 3 months, and treatment continued until progression or after two consecutive judgements of stable disease. Response rate was the main end point of the study. Of 141 patients eligible, 72 were randomised to 5-FU alone (arm A) and 69 to 5-FU + IFN (arm B). Responses were 9/72 (12.5%) in arm A and 6/69 (8.7%) in arm B; complete responses were three in arm A and two in arm B. Progression-free survival (median 4 months) and survival (median 12 months) were identical in the two arms. Toxicity was almost absent in arm A and moderate in arm B, represented mainly by haematological toxicity (usually leucopenia). In conclusion, overall survival was good in both arms of treatment and toxicity was moderate. While the response rate with 5-FU alone was in accord with the literature data, response to 5-FU + IFN was lower than expected. At least at this dosage and schedule, the association of 5-FU and IFN is no better than 5-FU alone and is of no clinical interest.


British Journal of Cancer (1996) 74, 971-974
© 1996 Stockton Press All rights reserved 0007-0920/96 $12.00

### A phase II randomised trial of 5-fluorouracil with or without interferon alpha-2a in advanced colorectal cancer

A Piga, S Cascinu, L Latini, M Marcellini, M Bavosi, L Acito, R Bascioni, L Giustini, G Francini, A Pancotti, G Rossi, M Del Papa, F Carle and R Cellerino

*Medical Oncology and Statistics, University of Ancona; Medical Oncology of Fermo, Senigallia, Ascoli Piceno, Siena, Iesi; General Surgery of Senigallia and Civitanova, Italy.*

**Summary** With the association of 5-fluorouracil (5-FU) and alpha-interferon (IFN), objective responses as high as 26-63% have been reported in untreated patients with advanced colorectal cancer. However, grade 3-4 toxicity has also been reported. We have conducted a prospective phase II randomised study comparing 5-FU to 5-FU+IFN, to investigate whether the addition of IFN to a weekly 5-FU regimen devoid of significant toxicity used at our institutions could improve the effectiveness of 5-FU while maintaining acceptable toxicity. Patients with histologically proven advanced colorectal carcinoma were randomised to receive 5-FU 500 mg $m^{-2}$ intravenous (i.v.) bolus on days 1-5 followed by 5-FU 500 mg $m^{-2}$ i.v. bolus weekly from day 15, with or without IFN alpha-2a intramuscularly (i.m.) 1.5 mU daily on days 6-12 and 3 mU i.m. daily thereafter. The treatment was administered on an outpatient basis. Response was evaluated every 3 months, and treatment continued until progression or after two consecutive judgements of stable disease. Response rate was the main end point of the study. Of 141 patients eligible, 72 were randomised to 5-FU alone (arm A) and 69 to 5-FU+IFN (arm B). Responses were 9/72 (12.5%) in arm A and 6/69 (8.7%) in arm B; complete responses were three in arm A and two in arm B. Progression-free survival (median 4 months) and survival (median 12 months) were identical in the two arms. Toxicity was almost absent in arm A and moderate in arm B, represented mainly by haematological toxicity (usually leucopenia). In conclusion, overall survival was good in both arms of treatment and toxicity was moderate. While the response rate with 5-FU alone was in accord with the literature data, response to 5-FU+IFN was lower than expected. At least at this dosage and schedule, the association of 5-FU and IFN is no better than 5-FU alone and is of no clinical interest.

**Keywords:** fluorouracil; interferon; colorectal neoplasm; clinical trial; antineoplastic agent

There is presently no standard treatment for advanced colorectal cancer. Treatment with 5-FU induces response rates of around 15-20%, while attempts at modulating 5-FU activity, or using aggressive combination chemotherapy, might result in an increased response rate but barely affect survival.

With the association of 5-FU and alpha-interferon (IFN) objective responses in 13/17 untreated patients were initially reported (Wadler *et al.*, 1989). Response rates ranging from 26% to 63% have subsequently been obtained in other trials (Kemeny *et al.*, 1990; Wadler *et al.*, 1991; Padzur *et al.*, 1993); however, severe toxicity (grade 3-4) with the combination was also reported, including diarrhoea, neurolgical complications and toxic deaths. Futhermore, the impact of this combination on survival is not clear.

In October 1990 we started a prospective phase II randomised study comparing 5-FU with 5-FU+IFN in advanced, untreated colorectal carcinoma, with the aim of evaluating the effect on response and survival of the addition of IFN to a weekly 5-FU regimen of limited toxicity commonly administered on an outpatient basis at our institutions.

### Patients and methods

Patients with histologically proven advanced colorectal cancer, evaluable disease, age ≤75 years, performance status ECOG 0-1, life expectancy of 3 months or more, and no previous chemotherapy, were randomised to receive 5-FU mg $m^{-2}$ i.v. bolus (15 min infusion in saline) on days 1-5 and then weekly from day 15 (arm A); or 5-FU+IFN alpha-2a i.m. 6 mU $day^{-1}$ (arm B); IFN was started at day 6 and given at half dose for the first week. The initially planned IFN dose of 6 mU $day^{-1}$ was abandoned after 4 months of study, as a result of both systemic and haematological toxicity registered in the first patients, and a dose of 3 mU $day^{-1}$ was used in the following patients. Response was evaluated every 3 months and treatment continued until progression or after two consecutive judgments of stable disease. Staging and follow-up clinical assessment included haematochemistry, tumour markers, abdominal computed tomography (CT) or ultrasound, chest radiograph, plus other tests depending on the site of involvement. Oral informed consent was a prerequisite for study entry.

Evaluation of response and toxicity was by standard criteria (Miller *et al.*, 1981). The following dose adjustments were applied: 5-FU was delayed by 1 week and IFN was given at half dose for WBC between 2 000 and 3 000 or platelets between 75 000 and 100 000; for lower values, 1 week delay for both drugs was required. Paracetamol 500-1000 mg orally was used in association with IFN, usually limited to the first weeks of treatment.

The estimated sample size was of 82 patients per arm, sufficient to show a difference of response rate from 15% (expected from 5-FU alone) to 35% (5-FU+IFN) at a significance level of 0.05 with a power of 80%. Statistical evaluation was accomplished by intention-to-treat analysis. The characteristics of patients in arm A and B were compared by the Wilcoxon test (age and time from diagnosis) and by the chi-square test (sex, site of primary, performance status). Overall response rates were compared by the chi-square test. Survival curves were estimated by the Kaplan-Meier method (Kaplan and Meier, 1958) and compared by the log-rank test (Peto and Peto, 1972).

### Results

A total of 142 patients were randomised from October 1990 to December 1993. At that time, response rate and its confidence limits in arm B were shown to be inferior to those

Correspondence: A Piga, Medical Oncology, University of Ancona, Ospedale Torrette, 60020 Ancona, Italy
Received 19 January 1996; revised 1 April 1996; accepted 19 April 1996

in arm A and judged to be of no clinical interest and accrual was stopped. Of the 142 patients enrolled, one patient was found to be ineligible since further diagnostic work-up following randomisation showed focal liver lesions, previously interpreted as metastases, to be benign lesions. This patient is still alive and with no evidence of disease (NED) 30 months after randomisation. All other patients are included in the analysis and evaluated by intention-to-treat. Of 141 eligible patients, 88 were male and 53 female, with a median age of 62 years. Primary site was colon in 104 cases and rectum in 37 cases.

Seventy-two patients were randomised to arm A and 69 patients were randomised to arm B; the characteristics of the two groups were comparable (Table I), with no statistically significant difference found (not shown). Approximately 25% of patients had significant cancer-related symptoms (19/72 in arm A, 18/69 in arm B), while the vast majority of patients were asymptomatic.

Protocol deviations were: two patients randomised to arm B were treated with aggressive platinum-based chemotherapy, since the origin of their tumour from the intestinal tract was not accepted as conclusive by the responsible physicians; one of these patients had a partial response (PR) followed by progression (P) at 26 months and is still alive 40 months from the start of treatment. One patient per arm over the age of 75 and in good general condition was randomised and evaluated, as well as two patients per arm with performance status ECOG 2 (Karnofsky 60).

Responses were 9/72 (12.5%) in arm A and 6/69 (8.7%) in arm B; 95% confidence limits showed wide overlap between the two groups (Table II). The difference in response rates was 3.8% in favour of 5-FU alone; the 95% confidence interval for this difference was −8.4% to 16.0%. An advantage in favour of the combination of 5-FU+IFN higher than 8.4% can therefore be confidently excluded at the 95% level based on these data.

Complete responses (CR) were three in arm A and two in arm B. Progression-free survival (median 4 months) and survival (median 12 months) were identical in the two arms (Figures 1 and 2). Duration of response was 5–15 months in arm A; in arm B, four patients survived without progression for more than 20 months. Two of them were CR patients with disease involving liver and distant nodes, and liver and bone respectively; both of them are still alive with NED at 41 and 36 months; another patient with liver involvement had stable disease (SD), still stable at 23 months; the fourth was one of the patients treated with an aggressive platinum-containing regimen, since an ovarian origin of the tumour could not be completely ruled out; this patient had a partial response and progressed after 26 months.

Nine patients in arm A and 5 patients in arm B were not evaluable for response (and respectively nine and seven for toxicity). Reasons for non-evaluability were: refusal to continue treatment beyond 1 month (seven patients in arm A, one patient in arm B), treatment received different from treatment planned (two patients in arm A, three patients in arm B), early cessation of treatment for cardiac ischaemia (one patient in arm B).

**Table I** Characteristics of the patients

| | *Arm A (5-FU)* | *Arm B (5-FU+IFN)* |
|---|---|---|
| Number of patients | 72 | 69 |
| Age (years) | | |
| Median | 61.5 | 62.9 |
| Range | 34–78 | 33–77 |
| Sex (M/F) | 47/25 | 41/28 |
| Site of primary (colon/rectum) | 54/18 | 50/19 |
| Time from diagnosis to treatment (months) | | |
| Median | 2.6 | 2.7 |
| Range | 0–129 | 0–96 |
| Performance status (ECOG) | | |
| 0 | 53 | 51 |
| 1 | 17 | 16 |
| 2 | 2 | 2 |
| Number of sites involved | | |
| 1 | 57 | 55 |
| 2 | 9 | 10 |
| 3 | 6 | 4 |
| Maximum extension of disease | | |
| Abdominopelvic | 16 | 10 |
| Hepatic | 39 | 40 |
| Extraabdominal | 17 | 19 |

**Table II** Response to treatment

| | *Arm A (5-FU)* | *Arm B (5-FU+IFN)* |
|---|---|---|
| Number of patients | 72 | 69 |
| Patients evaluable for response | 63 | 64 |
| Complete responses (CR) | 3 | 2 |
| Partial responses (PR) | 6 | 4 |
| Overall response | 9/72 | 6/69 |
| percentage | 12.5 | 89.7 |
| (95% confidence limits of the percentage) | (4.9–20.1) | (2.0–15.3) |
| Median time to progression (months) | 4 | 4 |
| Median survival (months) | 12 | 12 |

**Figure 1** Progression-free survival curves for the two arms, calculated from date of randomisation to first progression. Kaplan–Meier estimates (—), 5-FU ($n$=72); (- - - -), 5-FU+IFN ($n$=69).

**Figure 2** Survival curves for the two arms, calculated from date of randomisation to death. Kaplan–Meier estimates. (———), 5-FU ($n$=75); (- - - -), 5-FU+IFN ($n$=69).

Response by site of involvement is depicted in Table III. The most frequently involved site was the liver, where responses were observed in approximately 10% of patients; response rate was higher for nodal involvement.

Toxicity was almost absent in arm A and moderate in arm B, represented mainly by haematological toxicity, usually leucopenia (Table IV). In the initial part of the study, with IFN at 6 mU day$^{-1}$, systemic (fever, flu-like syndrome) and haematological toxicities were more severe and did not allow patients to receive the planned treatment in seven of eight instances; the eighth patient refused to double the dose from 3 mU to 6 mU. Grade 1–2 gastrointestinal toxicity was common and was mainly represented by transient diarrhoea, while mucositis, nausea and vomiting were infrequently observed; mild toxicity related to IFN (fever, flu-like syndrome) was common in arm B, usually limited to the first 1–2 weeks of treatment and controlled by oral paracetamol. The dose of 5-FU received was approximately 10% lower in arm B than in arm A (Table IV); IFN was given at approximately 80% of the planned dose.

Since response to 5-FU + IFN was low (with upper 95% confidence limit at 15%) and very unlikely to be influenced by completion of accrual (82 patients per arm planned), the overall result was judged of no interest, and the trial was stopped.

### Discussion

The treatment of advanced colorectal cancer traditionally relies on drugs and combinations devoid of signifcant toxicity. Attemps have been made recently towards more aggressive chemotherapy regimens. These have in general resulted in improved response rates; effects on survival are less clear. Most of the combinations employed are 5-FU-based regimens, in which the added drugs are biochemical modulators of the 5-FU effects (Peters and van Groningen 1992).

One of these recently introduced 5-FU modulators is α-interferon. After the experimental demonstration that IFN is able to increase the anti-tumour activity of 5-FU (Pfeffer and Tamm, 1984; Elias and Sandoval, 1989; Chu *et al.*, 1990; Danhauser *et al.*, 1993; Houghton *et al.*, 1993), several studies were performed in order to evaluate the role of the association of the two drugs in a clinical setting. Although the exciting results initially reported by Wadler *et al.* (1989) were not completely confirmed by others, a response rate of 30–40% was commonly observed; however, severe associated toxicity (mucositis, diarrhoea, leucopenia, neurological impairment and toxic deaths) was also reported (Kemeny *et al.*, 1990; Wadler *et al.*, 1991; Pazdur *et al.*, 1993), while the effect on survival is entirely unclear. In addition, optimal doses and schedules have yet to be identified.

The aim of the present study was to evaluate the contribution of IFN to the clinical activity of a weekly administration of 5-FU, devoid of relevant toxicity and given entirely on an outpatient basis, commonly employed at our institutions as palliative treatment of advanced colorectal cancer. The dosage of 5-FU was in the range of clinical effectiveness yet with low toxicity (500 mg m$^{-2}$ weekly); schemes of equivalent dose intensity have been reported effective and are currently used in clinical trials (Lokich *et al.*, 1989; Leichman *et al.*, 1995).

The dosage of IFN was the maximum allowed in our patients. A dose of 6 mU day$^{-1}$ (42 mU per week) was planned, but was shown in the first patients treated in our study not to be feasible; most of the patients received 3 mU day$^{-1}$.

The results obtained in our study showed a response rate to the combination similar to that obtained with 5-FU alone, with identical time to progression and overall survival. Several theorectical reasons might explain this failure of IFN to improve the effectiveness of 5-FU. One of these could be the use of a lower dose of IFN than that employed in the original scheme by Wadler *et al.* (1989). However, the few phase II studies employing low-dose IFN showed low toxicity and a response rate close to 30% (Botto *et al.*, 1991; Hansen *et al.*, 1993; Meehan *et al.*, 1993).

Another possible cause could be our choice of giving 5-FU by bolus injections (even in the loading course). However, on the basis of literature data (John *et al.*, 1993; Pazdur *et al.*, 1993; Leichman *et al.*, 1995), it is unlikely that such a difference in activity could be explained by this change of schedule only.

Contrasting results have been reported in randomised trials comparing 5-FU to 5-FU + IFN, with one study in which responses were higher with the combination (26.8% *vs* 10.1% with 5-FU alone) and also survival analysis showed a moderate but significant advantage in favour of the combination (Dreyfus *et al.*, 1995). In the majority of studies the 5-FU/IFN combination failed to show any therapeutic improvement compared with 5-FU alone, while the incidence and severity of toxicity was significantly higher (York *et al.*, 1993; Corfu-A Study Group, 1995; Hill *et al.*, 1995). Increase in incidence of toxic effects, without increase in response rates, was also recorded when IFN was added to a 5-FU/leucovorin combination in randomised trials (Seymour *et al.*, 1994; Kohne *et al.*, 1995).

Furthermore, in our study, despite the use of a combination of 5-FU and IFN of moderate toxicity, a dose reduction was often required for both drugs because of toxicity, and the attempts to use IFN at a higher dosage soon had to be abandoned.

**Table III** Response after 3 months, by site involvement

| | *Arm A (5-FU)* | | | *Arm B (5-FU+αIFN)* | | |
|---|---|---|---|---|---|---|
| | *No. of patients* | *No. evaluable* | *OR* | *No. of patients* | *No. evaluable* | *OR* |
| Primary tumour | 11 | 7 | 1 | 12 | 11 | 0 |
| Liver | 51 | 41 | 4 | 52 | 47 | 5 |
| Lung | 14 | 14 | 1 | 11 | 10 | 0 |
| Peritoneal | 6 | 6 | 2 | 5 | 5 | 1 |
| Skin | 3 | 1 | 0 | 4 | 4 | 0 |
| Bone | 2 | 2 | 1 | 5 | 3 | 1 |
| Distant nodes | 5 | 3 | 1 | 7 | 5 | 3 |

OR, odds ratio.

**Table IV** Dose intensity and leucopenia during the first 6 months of treatment

| | *Arm A (5-FU)* | | | | | *Arm B (5-FU+IFN)* | | | | | |
|---|---|---|---|---|---|---|---|---|---|---|---|
| | | | *Leucopenia* | | | | | | *Leucopenia* | | |
| | *No. of patients* | *%FU* | *Gr 1* | *2* | *3* | *No. of patients* | *%FU* | *%IFN* | *Gr 1* | *2* | *3* |
| Month 1 | 63 | 96 | 12 | 6 | 3 | 62 | 94 | 84 | 10 | 14 | 4 |
| Month 2 | 58 | 98 | 5 | 1 | 1 | 58 | 91 | 80 | 17 | 10 | 0 |
| Month 3 | 52 | 99 | 6 | 1 | 0 | 55 | 90 | 81 | 17 | 8 | 0 |
| Month 4 | 34 | 97 | 5 | 3 | 0 | 33 | 89 | 80 | 13 | 5 | 0 |
| Month 5 | 30 | 98 | 8 | 0 | 0 | 26 | 90 | 80 | 9 | 1 | 0 |
| Month 6 | 26 | 98 | 5 | 1 | 0 | 22 | 93 | 78 | 7 | 3 | 0 |

Gr, grade.

In conclusion, the addition of IFN to 5-FU at doses of the two drugs devoid of significant toxic effects did not result in improved efficacy with respect to 5-FU alone. As was recently stressed (Kemeny, 1995), the heterogeneity of the patients with advanced colorectal cancer might account for the variability of response rates and survival in this disease and the difficulty of obtaining univocal results in clinical trials. Since advanced colorectal carcinoma is a condition in which survival times seem to be independent from any presently available medical treatment, the attempts at improving response rates, by testing more toxic and expensive combinations should be limited to experimental clinical setting and avoided in routine clinical practice.

**Acknowledgements**
We thank the following physicians for entering their patients into the trial: Enrica Testa, Urbino; Gabriele Marchegiani, Tolentino; Augusto Marcosignori, Senigallia; Bruno Neri, Florence; Giorgio Di Rosa, Camerino; Germano Gabrielli, Iesi.

### References

BOTTO HG, GALVEZ C, PALAO MARCO F, BONAMASSA M, FABEIRO MA, MARIN F AND BOTTO IS. (1992). 5-fluorouracil (5-FU) and interferon alpha 2b (IFN) in advanced colorectal cancer: results in 47 patients (abstract 519). *Proc. Am. Soc. Clin. Oncol.*, **11**, 176.

CHU E, ZIN S, BOARMAN D AND ALLEGRA CJ. (1990). Interaction of gamma interferon and 5-fluorouracil in the H630 human colon carcinoma cell line. *Cancer Res.*, **50**, 5834–5840.

CORFU-A STUDY GROUP. (1995). Phase III randomised study of two fluorouracil combinations with either interferon alfa-2a or leucovorin for advanced colorectal cancer. *J. Clin. Oncol.*, **13**, 921–928.

DANHAUSER LL, FREIMANN JH, GILCHRIST TL, GUTTERMAN JU, HUNTER CY, YEOMANS AC AND MARKOWITZ AB. (1993). Phase I and plasma pharmacokinetic study of infusional fluorouracil combined with recombinant interferon alpha-2b in patients with advanced cancer. *J. Clin. Oncol.*, **11**, 751–761.

DREYFUS B, DUFOUR P, HUSSEINI F, CURE H, OLIVIER JP, DUMAS F, PREVOT G, MARTIN C, DUCLOS B, THILL L, AUDHUY B, BERGERAT JP AND OBERLING F. (1995). Randomised study of 5-fluorouracil (5 FU) versus 5 FU+alpha 2 A interferon (IFN as treatment for metastatic colorectal carcinoma (MCRC) (abstract O262). In *Proceedings of the 5th International Congress on Anticancer Chemotherapy*, p 113.

ELIAS L AND SANDOVAL JM. (1989). Interferon effects upon fluorouracil metabolism by HL60 cells. *Biochem. Biophys. Res. Commun.*, **163**, 867–874.

HANSEN R, SCHUETZ M, VUKELICH M, BLAKE D AND ANDERSON T. (1991). A phase II study of 5-fluorouracil (5FU) infusion, interferon alpha, and dipyridamole in advanced colorectal cancer (abstract 481). *Proc. Am. Soc. Clin. Oncol.*, **10**, 154.

HILL MH, NORMAN A, CUNNINGHAM D, FINDLAY M, WATSON M, NICOLSON V, WEBB A, MIDDLETON G, AHMED F, HICKISH T, NICHOLSON M, O'BRIEN M, IVESON T, IVESON A AND EVANS C. (1995). Royal Marsden phase III trial of fluorouracil with or without interferon alfa-2b in advanced colorectal cancer. *J. Clin. Oncol.*, **13**, 1297–1302.

HOUGHTON JA, MORTON C, ADKINS D AND RAHMAN A. (1993). Locus of the interaction among 5-fluorouracil, leucovorin and interferon-α2a in colon carcinoma cells. *Cancer Res.*, **53**, 4243–4250.

JOHN WJ, NEEFE JR, MACDONALD JS, CANTRELL J AND SMITH M. (1993). 5-fluorouracil and interferon-alpha-2a in advanced colorectal cancer. Results of two treatment schedules. *Cancer*, **72**, 3191–3195.

KAPLAN EL AND MEIER P. (1958). Non-parametric estimation from incomplete observation. *J. Am. Stat. Assoc.*, **53**, 457–481.

KEMENY N. (1995). Chemotherapy for colorectal carcinoma: one small step forward, one step backward. *J. Clin. Oncol.*, **13**, 1287–1290.

KEMENY N, YOUNES A, SEITER K, KELSEN D, SAMMARCO P, ADAMS L, DERBY S, MURRAY P AND HOUSTON C. (1990). Interferon alpha-2a and 5-fluorouracil for advanced colorectal carcinoma: assessment of activity and toxicity. *Cancer*, **66**, 2470–2475.

KOHNE CH, WILKE H, HECKER H, SCHOFFSKY P, KAUFFER C, RAUSCHECKER H, ANDREESEN R, OHL U, LANGE HJ, KLAASSEN U, WESTERHAUSEN M, HIDDEMANN W, HENNEMANN B, SCHOTT G, BADE J, STROHMEYER G, HARSTRICK A, SCHUBERT U, BOKEMEYER C AND SCHMOLL HJ. (1995). Interferon-alpha does not improve the antineoplastic efficacy of high-dose infusional 5-fluorouracil plus folinic acid in advanced colorectal cancer. *Ann. Oncol.*, **6**, 461–466.

LEICHMAN CG, FLEMING TR, MUGGIA FM, TANGEN CM, ARDALAN B, DOROSHOW JH, MEYERS FJ, HOLCOMBE RF, WEISS GR, MANGALIK A AND MACDONALD JS. (1995). Phase II study of fluorouracil and its modulation in advanced colorectal cancer: a Southwest Oncology Group study. *J. Clin. Oncol.*, **13**, 1303–1311.

LOKICH JJ, AHLGREN JD, GULLO JJ, PHYLIPS JA AND FRYER JG. (1989). A prospective randomized comparison of continuous infusion fluorouracil with a conventional bolus schedule in metastatic colorectal cancer: a Mid-Atlantic Oncology Program study. *J. Clin. Oncol.*, **7**, 425–432.

MEEHAN K, SEIDLER C, GEHR G AND MAURER LH. (1993). An outpatient regimen of 5-fluorouracil (5-FU) and recombinant interferon alpha-2b (rIFNa-2B) in metastatic adenocarcinoma of the colon (MAC) (abstract 678). *Proc. Am. Soc. Clin. Oncol.*, **12**, 222.

MILLER AB, HOOGSTRATEN B, STAQUET M AND WINKLER A. (1981). Reporting results of cancer treatment. *Cancer*, **47**, 207–214.

PAZDUR R, AJANI JA, PATT YZ, GOMEZ J, BREADY B AND LEVIN B. (1993). Phase II evaluation of recombinant alpha-2a-interferon and continuous infusion fluorouracil in previously untreated metastatic colorectal adenocarcinoma. *Cancer*, **71**, 1214–1218.

PETERS GJ AND VAN GROENINGEN CJ. (1991). Clinical relevance of biochemical modulation of 5-fluorouracil. *Ann. Oncol.*, **2**, 469–480.

PETO R AND PETO J. (1972). Asymptotically efficient invariant procedures. *J. R. Stat. Soc. A.*, **135**, 185–206.

PFEFFER LM AND TAMM I. (1984). Interferon inhibition of thymidine incorporation into DNA through effects on thymidine transport and uptake. *J. Cell Physiol.*, **121**, 431–436.

SEYMOUR MT, SLEVIN M, CUNNINGHAM D, KERR D, JAMES R, LEDERMAN J, PERREN T, MCADAM W, DUFFY A, STENNING S AND TAYLOR I. (1994). A randomized assessment of interferon-α2a (IFNα) as a modulator of 5-fluorouracil (5FU) and leucovorin (LV) in advanced colorectal cancer (abstract 621). *Proc. Am. Soc. Clin. Oncol.*, **13**, 209.

WADLER S, SHCWARTZ EL, GOLDMAN M, LYVER A, RADER M, ZIMMERMAN M, ITRI L, WEINBERG V AND WIERNIK PH. (1989). Fluorouracil and recombinant alfa-2a-interferon: an active regimen against advanced colorectal carcinoma. *J. Clin. Oncol.*, **7**, 1769–1775.

WADLER S, LEMBERSKY B, ATKINS M, KIRKWOOD J AND PETRELLI N. (1991). Phase II trial of 5-fluorouracil and recombinant interferon alfa-2a in patients with advanced colorectal carcinoma: an Eastern Cooperative Oncology Group study. *J. Clin. Oncol.*, **9**, 1806–1810.

YORK M, GRECO FA, FIGLIN RA, EINHORN L, MAN T, COCKEY L, MOTT D AND LIGHT SE. (1993). A randomized phase III trial comparing 5-FU with or without interferon alfa 2a for advanced colorectal cancer (abstract 590). *Proc. Am. Soc. Clin. Oncol.*, **12**, 200.